# Reform progress and achievements of China’s incentive policies for pediatric medicine over the last decade

**DOI:** 10.3389/fphar.2025.1561095

**Published:** 2025-03-26

**Authors:** Qiwei Wang, Xing Ling, Zhengli Dai, Xiaoling Wang, Wen Guo, Guiliang Chen

**Affiliations:** ^1^ China State Institute of Pharmaceutical Industry, Shanghai, China; ^2^ China Pharmaceutical Industry Information Center, Shanghai, China; ^3^ Beijing Children’s Hospital, Capital Medical University, Beijing, China; ^4^ Shanghai Center for Drug Evaluation and Inspection, Shanghai, China

**Keywords:** pediatric medicine, incentive policies, accessibility, reform, progress and achievements

## Abstract

**Background:**

The accessibility of pediatric medicine is a global challenge. The issuance of the “Several Opinions on Ensuring the Use of Children’s Medicine” by six ministries in China in 2014 marked the formation of the policy framework. This study aims to systematically review the progress and achievements of incentive policies for China’s pediatric medicine.

**Methods:**

By analyzing policy documents, clinical trial data, review and approval results, medical insurance directories, volume-based procurement data, as well as adverse reaction reports, the implementation effects of incentive policies for China’s pediatric medicine were assessed.

**Results:**

China has made progress in legislation, research and development, review and approval, production, access, payment, and use of pediatric medicine. The number and variety of pediatric clinical trials have increased year by year. Some medicine on the Encouraged Research and Submission List of Pediatric Medicine have successfully entered the market. Priority review and approval policies have effectively facilitated the rapid approval of pediatric medicine. However, the availability still needs to be improved, especially in the field of medicine for young children (<6 years).

**Conclusion:**

Although incentive policies for China’s pediatric medicine have achieved favorable effects, the synergy of the policies still needs to be strengthened. It is recommended that the Chinese government place greater emphasis on the introduction of mandatory regulations and incentive policies, enhance the synergy between policies, use a combination of fiscal and medical insurance tools and follow up on the application of new technologies to comprehensively improve the accessibility of pediatric medicine in the future. This might be helpful for guaranteeing the safety, efficacy and economy of pediatric medicine.

## 1 Introduction

The accessibility of pediatric medicine is a global challenge. Such issues as the delayed update of pediatric drug instructions and the difficulty in conducting pediatric clinical trials have made off-label drug use common in children. A survey (Mei et al., 2019) in China found that nearly half of pediatricians and most pharmacists had engaged in off-label prescribing or dispensing, mainly due to the lack of child-friendly medicines. In China, home to approximately 253 million youth aged 0–14 (17.95% of the total population) (National Bureau of Statistics, 2021), which makes solving the problem of insufficient children’s medicines more urgent (Sun and Li, 2023). Similar challenges are not unique to China (Meng et al., 2022). However, countries such as the United States and the European Union have implemented legislation and guidelines to encourage pediatric drug development (Rose, 2021). China began to pay attention to this in 2011, but the policy framework formation started with the “Several Opinions on Ensuring the Use of Children’s Medicine” released by six ministries in 2014, which put forward relatively clear requirements from the pediatric medicine industry chain perspective (Yan and Shao, 2022). Since then, pediatric medicine development in China has entered a rapid growth period. This article aims to systematically sort out existing problems in pediatric medicine security, analyze incentive policies published, explore policy effects and discuss trends.

## 2 Materials and methods

### 2.1 Policy document data

Pediatric medicine-related policies were retrieved from official government websites and the China Pediatric Drug Database (http://cpd.pharmadl.com), using keywords such as “pediatrics,” “pediatric medicine,” “children” and “children’s medicine” and excluded irrelevant measures, work reports, reply summaries, statistical classifications and invalid documents. The search spanned 1 January 2014 to 31 December 2024.

### 2.2 Research and development phase data

Pediatric clinical trial data registered with the Center for Drug Evaluation (CDE), accessed via the global pediatric clinical trial registration module of the China Pediatric Drug Database cover subjects aged 0–17 years. All trials were publicized from 1 January 2014 to 31 December 2024.

### 2.3 Review and approval phase data

Annual drug review reports (2016–2023) were obtained from the National Medical Products Administration (National Medical Products Administration, 2024) via keyword searches. Analyzing the marketing status of drugs on the Encouraged Research and Submission List of Pediatric Medicine helps understand the impact of incentive policies. All the marketed pediatric drug varieties, excluding empirical pediatric medicine and withdrawn data, were sourced from the China Pediatric Drug Database (counted by generic name and dosage form), with a launch time before 31 December 2024. In this article, empirical pediatric medicine refers to drugs whose labels do not specify dosages for specific pediatric population but do not prohibit their use. Pediatric-specific medicines are those labeled exclusively for pediatric use (either treating pediatric conditions exclusively or providing only pediatric dosage details). Pediatric co-medicine refers to drugs with pediatric indications (not exclusive to children) and clearly specified pediatric dosages.

### 2.4 Payment phase data

The national medical insurance drug directory (2024 edition) and the national volume-based (VBP) procurement drug directory were obtained from the National Healthcare Security Administration (NHSA) and the Shanghai Medical Procurement Agency (SMPA) respectively. Pediatric drugs in these directories were identified by comparing generic names and dosage forms with those in the China Pediatric Drug Database. The comparison focused on drugs launched before 31 December 2024.

### 2.5 Post-market research phase data

The National Adverse Drug Reaction Monitoring Annual Reports (2014–2023) were obtained from the NMPA using the keyword “adverse drug reaction monitoring annual report” and pediatric adverse reaction data were extracted from the reports.

**FIGURE 1:**
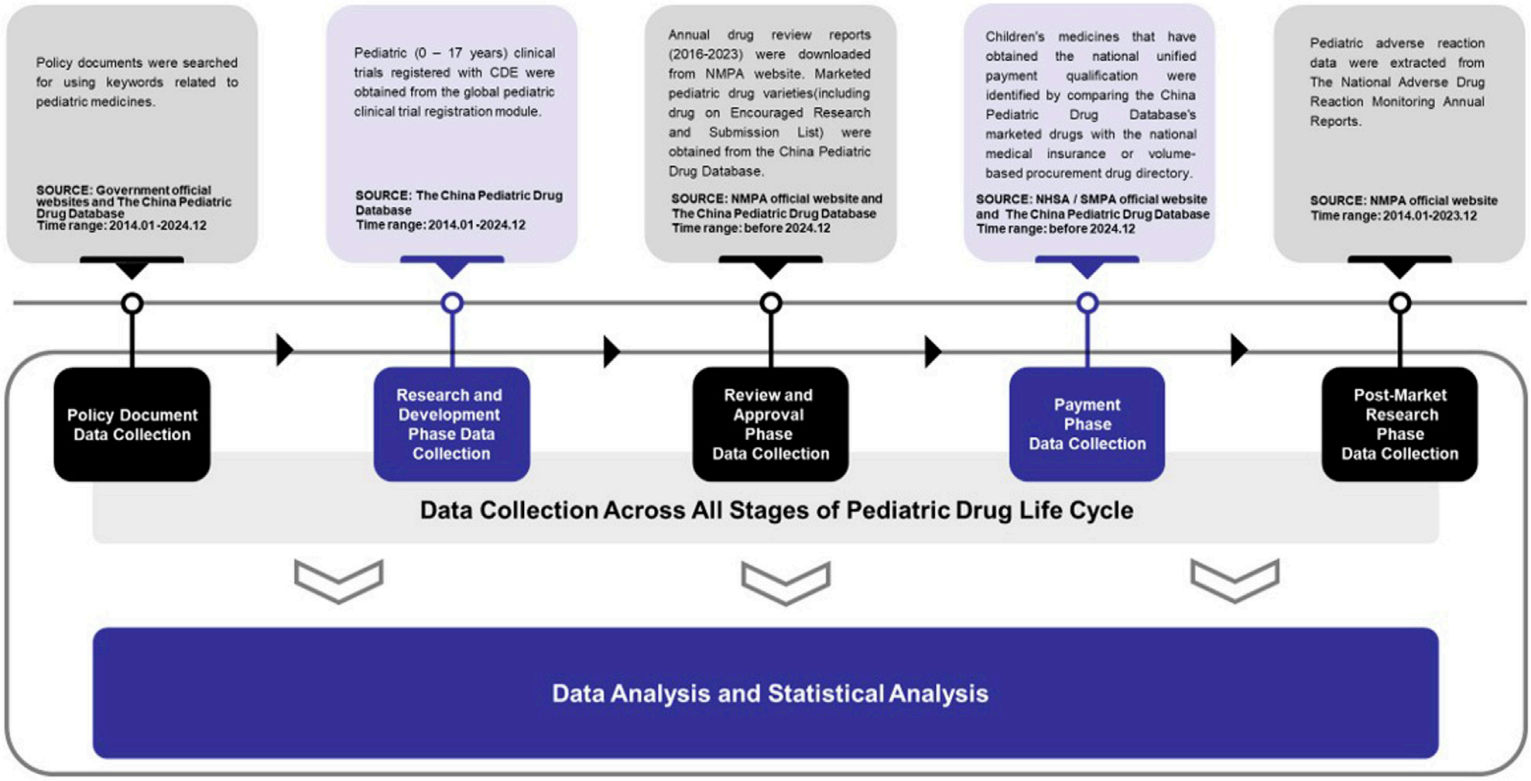
Flowchart of data collection and inclusion criteria.

Completing this work involved data analysis and statistical analysis. Microsoft Excel software was used for data analysis (Figure 1).

## 3 Results

### 3.1 Issues faced in pediatric medicine security

#### 3.1.1 Lack of specific legislation for pediatric medicine

Currently, China lacks regulations specifically for pediatric medicine, which restricts the research and development as well as the launch of children -appropriate pharmaceuticals. Although the Constitution of the People’s Republic of China and the Protection of Minors Law provide basic legal protection for children, specific laws or regulations specifically for pediatric medicine are not well-established. The Drug Administration Law revised in 2019, included “pediatric medicine” for the first time, laying the foundation for the legalization of pediatric medicine. However, more time and incentive policies are still needed to fully implement these regulations (Chen et al., 2020).

#### 3.1.2 Pediatric drug shortages and labeling deficiencies

The availability of pediatric-specific drugs is severely restricted. Currently, pediatric-specific medications account for only about 3% of all marketed drugs and are predominantly found in categories such as antipyretics, analgesics, anti-inflammatory agents, antimicrobials, and vitamin/mineral supplements. Notably, there is a glaring absence of pediatric-specific drugs in critical therapeutic domains like antineoplastic agents, anesthetics and antidotes (Wei et al., 2024).

The design of drugs intended for both pediatric and adult use typically prioritizes adult requirements, leading to an even more pronounced scarcity of dosage forms and specifications that are appropriate for children (Qin et al., 2021). Moreover, drug labeling information for pediatric medicines is highly inadequate. Essential details such as pharmacological properties, toxicology, clinical trial data, safety information and clear guidelines on dosage and administration are often missing. These omissions can have a detrimental impact on the safety and efficacy of pediatric medications (Zhang et al., 2023).

Due to these shortages and labeling deficiencies, clinicians are frequently compelled to use drugs off-label. This practice, which involves administering drugs beyond the recommended dosage, indications, or target populations specified in the drug labeling, is widespread while the potential risks cannot be ignored.

#### 3.1.3 High research and development difficulty and small market capacity

The research and development of pediatric drugs is relatively difficult and costly. On one hand, pediatric drugs have special requirements in terms of dosage forms, excipients and additives (Wang and Chen, 2024). On the other hand, clinical trials for pediatric drugs are challenging due to difficulties in recruiting subjects, a shortage of pediatric clinical trial institutions and a relative lack of professional clinical researchers (Zhao et al., 2017).

#### 3.1.4 Immature production and distribution system and information sharing

The demand for pediatric drugs often presents as small quantities with regional dispersion and is significantly affected by seasonal changes. As a result, manufacturers are often unwilling to produce these drugs, suppliers are hesitant to stock and distribution enterprises show limited motivation to distribute them (Du et al., 2018). Additionally, due to the regional dispersion of demand, the cost for manufacturers to obtain market information is high and medical institutions are often unclear about which enterprises have production capabilities. These factors have led to shortages of clinically essential pediatric drugs, low distribution rates and insufficient availability and accessibility. These issues are particularly prominent in children’s specialty hospitals, maternal and child health hospitals and tertiary hospitals.

#### 3.1.5 Unreasonable prescriptions and high incidence of adverse reactions

Unreasonable prescriptions for pediatric medicine are quite common, including improper selection of indications, errors in dosing frequency or route and overdose use. These issues not only affect therapeutic effects but also increase the risk of adverse reactions. The incidence of adverse drug reactions/events in pediatric medicine is high and the proportion of serious adverse drug reactions/events has been increasing year by year (Cui et al., 2021).

#### 3.1.6 Insufficient post-marketing surveillance and evaluation

Post-marketing surveillance and evaluation of pediatric medicine are relatively insufficient, lacking a dedicated monitoring and evaluation mechanism for the marketed pediatric medicine. This is crucial for controlling drug risks and promoting the development of rational clinical medicine use (Yue and Li, 2017).

### 3.2 Incentive policies for pediatric medicine

In 2014, the former National Health and Family Planning Commission and five other departments jointly issued the “Several Opinions on Ensuring the Use of Children’s Medicine” which for the first time established a policy framework for the supply of pediatric medicine. It put forward specific requirements for ensuring pediatric medicine from several aspects, including encouraging research and development, accelerating review and approval, ensuring production and supply, strengthening quality supervision, promoting rational medicine use, improving policy system construction and enhancing comprehensive capabilities. Since then, policy reforms have evolved from initial establishment to continuously enriching connotations, gradually improving system content and now entering a new stage of strengthening policy coordination and transitioning into a policy environment.

From 2014, a total of 51 incentive policies for pediatric medicine were identified through retrieval and collection. These policies were categorized according to different stages of the pharmaceutical lifecycle (Supplementary Table S1), including 28 in the R&D stage, 7 in the approval stage, 4 in the production stage, 3 in the market access stage, 3 in the payment stage and 6 in the utilization stage. Support measures include establishing technical guidelines, developing procedures to increase pediatric information in drug labels, creating a fast-track approval process, offering R&D funding, providing a list of market-deficient drugs, strengthening drug production and supply and establishing pediatric drug prescribing norms. The majority of these policies (68%) are concentrated in the R&D and approval stages, with the highest number (16) being technical guidelines for R&D. In contrast, there are relatively fewer policies on standardizing prescribing behavior, providing funding support.

In the R&D phase: The government has increased funding for pediatric medicine R&D to promote the development of pediatric medicine varieties and key technologies. A 6-year data protection period is granted for pediatric-specific drug trial data. For the first approved pediatric-specific new varieties, dosage forms and specifications, as well as those that add pediatric indications or dosage and administration instructions, a market exclusivity period of up to 12 months is granted. These measures strengthen the incentive and protection of pediatric medicine intellectual property rights. A series of technical guidelines have been issued to standardize the research and development of pediatric medicine, covering aspects such as drug discovery, preclinical research, clinical research, and specialized disease research. Communication and management procedures have been established to intervene early, guide and improve the quality and efficiency of communication, thereby accelerating pediatric medicine R&D.

In the review and approval phase: The government has accelerated the review and approval of pediatric medicine, established a dedicated pathway for pediatric medicine review and approval and refined the principles for prioritizing pediatric medicines, including new varieties, dosage forms and specifications suitable for children’s physiological characteristics, as well as those that are in short supply in the market and encouraged for research and submission. The government has continuously optimized policy measures oriented by clinical needs.

In the production phase: Enterprises are guided to focus on addressing the shortage of pediatric medicine and the weak supply guarantee of low-priced pediatric medicine. A drug supply warning mechanism has been established to timely grasp the dynamics of short pediatric medicine, actively coordinate to resolve prominent issues and difficulties faced by enterprises and enhance production and supply capabilities. In terms of drug quality, safety, efficacy and quality controllability rigorous reviews must be conducted. Strict supervision must be implemented throughout the entire process of production, distribution and use.

In the access phase: The procurement approach has been optimized. Pediatric specialty non-patent drugs are directly procured through online listing. For pediatric drugs with clear pediatric indications, dosage and administration instructions, hospitals can expand the scope of available medicines without being limited by the “one drug, two specifications” rule (medical institutions can purchase no more than two types of the same dosage form for drugs with the same generic name) or the total number of drug varieties.

In the payment phase: The role of medical insurance in ensuring pediatric medicine is leveraged by timely including pediatric-appropriate dosage forms and specifications in the basic medical insurance payment scope. Drugs on the Encouraged Research and Submission List of Pediatric Medicine can be prioritized for inclusion in the medical insurance drug directory.

In the usage phase: For drugs that have been used clinically for many years but lack pediatric medicine data in their instructions, enterprises are encouraged to use data extrapolation and real-world data to complete and update instruction information for addressing the lag in information supplementation and the problem of off-label use in clinical practice. In terms of prescription standards, medical institutions are required to standardize prescriptions according to guidelines, promote information management and improve the level of rational medicine use. For the comprehensive clinical evaluation capacity, pediatric medicine evaluation plans are prioritized. Evaluation systems are established based on advantaged hospitals, evaluation technical guidelines are issued, adverse reaction monitoring and re-evaluation are strengthened and the scientific, rational, and safe use of pediatric medicine is fully ensured.

### 3.3 The implementation effects of incentive policies for pediatric medicine

#### 3.3.1 A significant increase in the number of pediatric clinical trials

**FIGURE 2:**
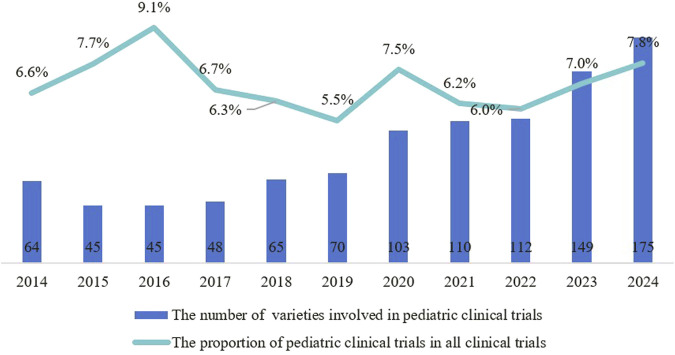
Trends in the number and proportion of pediatric clinical trials among all clinical trials from 2014 to 2024.

**FIGURE 3:**
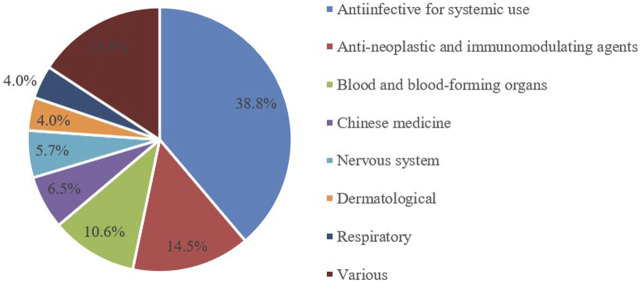
Distribution of ATC classifications for pediatric clinical trial drugs, 2014-2024.

From 2014 to 2024, a total of 1403 pediatric clinical trials were conducted, representing 5.1% of all clinical trials. The number of trial drug varieties in pediatric clinical trials grew from 64 in 2014 to 175 in 2024, at a compound annual growth rate of 10.7% (Figure 2). Pediatric clinical trial drugs were mainly classified under anti-infectives for systemic use (38.8%), followed by anti-neoplastic and immunomodulating agents (14.5%) and drugs for the blood and blood-forming organs (10.6%) (Figure 3). There has been an increase in pediatric clinical trials and the variety of trial drugs over the decade, indicating growing attention and progress in pediatric drug research.

#### 3.3.2 Encouraged research and submission list and priority review and approval process have accelerated the market approval of pediatric medicine

**FIGURE 4:**
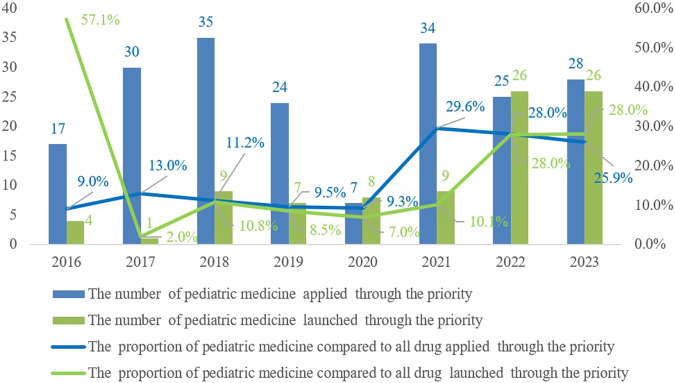
Trends in the number and proportion of pediatric medicine applied and launched through the priority review and approval process from 2016 to 2023.

China’s priority review and approval process commenced in 2016. By the end of 2023, the number of pediatric drug applications included in the priority review has shown an overall upward trend, with a total of 200 applications being admitted into the priority review channel. Among these applications, 90 pediatric drug varieties have successfully been approved and launched through the priority approval process (Figure 4), demonstrating the regulatory authorities’ emphasis on support for pediatric drug development.

Since June 2016, the state has published five batches of the Encouraged Research and Submission List of Pediatric Medicine, involving 170 drug specifications. As of now, 38 specifications across the first four batches are on the market, with 20 types approved through the priority review and approval process (Table 1). This policy has significantly accelerated the approval of pediatric medicines, reflecting a strong commitment to supporting their development and addressing clinical needs.

**TABLE 1:** Drug specifications have been marketed on encouraged research and submission list of pediatric medicine.

Batch No.	Time of batch release^a^	Directory No.	Generic name	Dosage forms	Specifications	Whether approved through priority review and approval process^b^
Batch 1	2016.06	6	Haloperidol	Oral Solution	1 mL:2 mg	
Batch 1	2016.06	10	Midazolam	Oral Solution	1 mL:5 mg	√
Batch 1	2016.06	11	Nimodipine	Oral Solution	1 mL:3 mg	
Batch 1	2016.06	14	Sodium Bicarbonate	Injection	1.4%(100 mL)	
Batch 1	2016.06	15	Chloral Hydrate	Enema		√
Batch 1	2016.06	16	Diazoxide	Oral Solution	30 mL:1.5 g	√
Batch 1	2016.06	18	Melphalan	Injection	50 mg	√
Batch 1	2016.06	21	Ursodeoxycholic Acid	Suspension	1 mL:50 mg	√
Batch 1	2016.06	22	Levetiracetam	Injection	5 mL:0.5 g	√
Batch 1	2016.06	27	Glucagon	Injection	1 mg	
Batch 2	2017.05	10	Clobazam	Tablet	10 mg	√
Batch 2	2017.05	11	Procarbazine	Capsule	50 mg	
Batch 2	2017.05	13	Vigabatrin	Oral Powder	0.5 g	√
Batch 2	2017.05	18	Mesalazine	Rectal Suppository	0.5 g	
Batch 2	2017.05	19	Desmopressin	Oral Solution	1 mL:0.36 mg	
Batch 2	2017.05	21	Atropine	Ophthalmic Solution	0.1%	√
Batch 2	2017.05	23	Baclofen	Oral Solution	1 mg/mL	
Batch 2	2017.05	24	Amlodipine	Suspension	1 mL:1 mg	
Batch 2	2017.05	25	Calcitriol	Oral Solution	1 mL:1 μg	
Batch 2	2017.05	27	Sulfamethoxazole + Trimethoprim, Combinations	Injection	1 mL: sulfamethoxazole 80 mg, trimethoprim 16 mg	
Batch 2	2017.05	29	Nitric Oxide	Gas		√
Batch 2	2017.05	31	Eculizumab	Injection	10 mg/mL	√
Batch 2	2017.05	35	Velaglucerase Alfa	Powder for Injection	400IU	
Batch 3	2019.08	5	Enalapril	Oral Solution	150 mL:0.15 g	
Batch 3	2019.08	11	Sapropterin	Tablet	0.1 g	
Batch 3	2019.08	13	Tacrolimus	Granules	1 mg	√
Batch 3	2019.08	13	Tacrolimus	Granules	0.2 mg	√
Batch 3	2019.08	14	Anakinra	Injection	0.67 mL:0.1 g	
Batch 3	2019.08	17	Dexmedetomidine	Nasal Spray	1 g:0.2 mg	
Batch 3	2019.08	22	Voriconazole	Suspension	1 mL:40 mg	
Batch 3	2019.08	25	Trientine	Capsule	0.25 g	√
Batch 3	2019.08	32	Sodium Phenylacetate + Sodium Benzoate, Combinations	Injection	50 mL	√
Batch 4	2023.08	10	Ganaxolone	Suspension	1 mL:50 mg	√
Batch 4	2023.08	12	Avalglucosidase Alfa	Injection	0.1 g	
Batch 4	2023.08	15	Glycerol Phenylbutyrate	Oral Solution	1 mL:1.1 g	√
Batch 4	2023.08	18	Turoctocog Alfa Pegol	Injection	1000IU	√
Batch 4	2023.08	18	Turoctocog Alfa Pegol	Injection	500IU	√
Batch 4	2023.08	22	Minocycline	Topical Foam	4%	√

Note:

^a^ The fifth batch release time is August 2024. As no drugs of this batch have been launched on the market so far, it is excluded from this table.

^b^ All drugs in the table are marketed and those with the tick box were approved through priority review and approval process, while those without the tick box were not.

#### 3.3.3 The quantity of pediatric drugs has increased, but their proportion relative to all medications has experienced a slight decline

**FIGURE 5:**
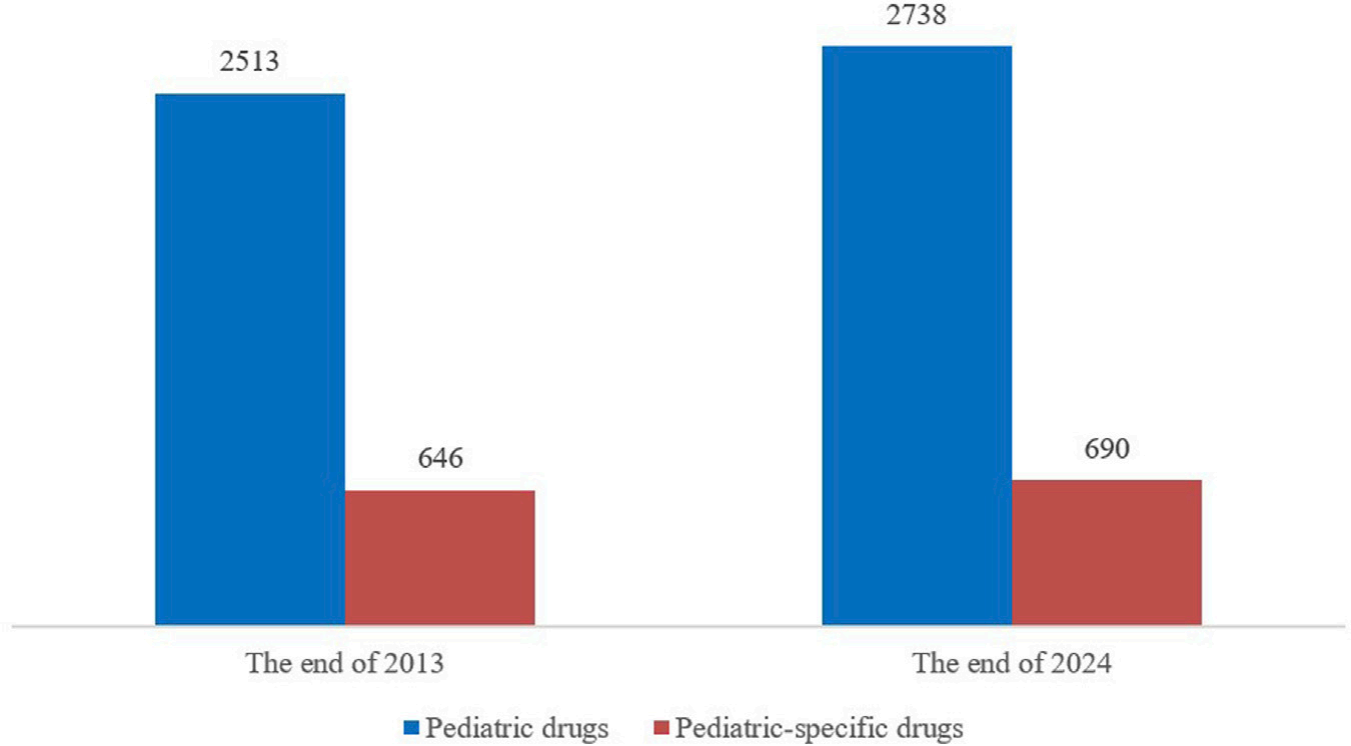
Comparison in the number of pediatric drugs and pediatric-specific drugs on the market in 2013 and 2024.

By the end of 2013, China had approved 2513 varieties of pediatric drugs for marketing, including 646 specifically formulated for children. By the end of 2024, the total had increased to 2738, marking an addition of 225 varieties compared to 2013 and pediatric-specific drugs rose to 690, up 44 from 2013. The proportion of pediatric-specific drugs among all pediatric drugs slightly decreased from 25.7% to 25.2% (Figure 5). Although the variety of medications for children is increasing, the proportion of pediatric-specific drugs has not significantly improved.

Newly approved pediatric drugs from 2014 to 2024 are mainly classified under anti-infectives for systemic use (33.2%), respiratory system agents (17.1%) and nervous system agents (14.4%) (Table 2). Notably, among newly approved pediatric-specific drugs, nervous system drugs had the highest number of approvals (34, 26.5%), followed by respiratory system agents (25.2%) and anti-infectives for systemic use (14.5%). This suggests that the development of pediatric-specific nervous system drugs have received greater attention and priority in the field of pediatric medication research.

**TABLE 2:** The ATC classification of newly approved pediatric drugs from 2014 to 2024.

ATC classification	Proportion	ATC classification	Proportion
Antiinfective for systemic use	33.2%	Cardiovascular system	3.2%
Respiratory system	17.1%	Musculoskeletal system	3.0%
Nervous system	14.4%	Systemic honnones (excluding sex homonesand insulin) preparations	1.7%
Alimentary tract and metabolism	7.3%	Dermatological	1.4%
Miscellany	5.8%	Traditional Chinese medicine	1.0%
Blood and blood forming organs	5.9%	Various	0.9%
Antineoplastic and immunomodulating agents	5.0%		

#### 3.3.4 Pediatric drugs inclusion in NRDL and VBP expands but remains underrepresented

National Reimbursement Drug List (NRDL) (2024 edition) included 5237 drug varieties (excluding traditional Chinese medicine decoctions), with 966 pediatric co-medicine varieties (18.4% of the total) and 170 pediatric-specific drug varieties (3.2% of the total). Compared to the 2017 edition, pediatric co-medicine varieties increased by 75 (with a 0.4% proportion reduction) and pediatric-specific drugs increased by 40 (with a 0.5% proportion increase). The VBP covered 456 varieties, including 159 pediatric co-medicine varieties (34.9% of the total) and 5 pediatric-specific drug varieties (1.1% of the total) (Table 3). Although the number of pediatric medications included in both directories has increased, their proportion remains relatively low, with the coverage of pediatric-specific drugs being particularly insufficient.

**TABLE 3:** National-level VBP included a total of 5 pediatric-specific drug varieties.

Generic name	Dosage forms	Specifications
Caffeine	Injection	1 mL:20 mg
Montelukast	Chewing Tablet	4 mg
Montelukast	Chewing Tablet	5 mg
Montelukast	Granules	0.5 g:4 mg
Atomoxetine	Capsule	25 mg
Atomoxetine	Capsule	10 mg
Atomoxetine	Oral Solution	100 mL:0.4 g

#### 3.3.5 Continued vigilance on pediatric adverse drug reactions

**FIGURE 6:**
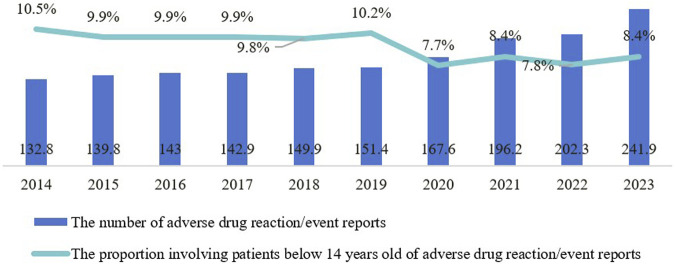
Trends in the number and proportion of pediatric-related adverse drug reaction reports from 2014 to 2023.

From 2014 to 2023, there was a year-on-year increase in adverse drug reaction/event reports received by the national monitoring network, rising from 1.328 million to 2.419 million (Figure 6). The proportion of reports involving patients under 14 averaged 8.7% during this period, peaking at 10.5% in 2014, then declining before rebounding to 8.4% in 2021 and 2023. Although the proportion of pediatric adverse drug reaction reports has fluctuated, the continuous increase in their absolute number highlights the severity of pediatric medication safety issues, which still require significant attention and further improvement.

## 4 Discussion

### 4.1 The absence of mandatory regulations hinders the substantial improvement of pediatric medicine supply and safety

Currently, China lacks specific laws for pediatric medicine. Experiences from some countries suggest that mandatory regulations may be more effective than incentive policies in promoting pediatric drug research and development. Mandatory regulations can directly encourage companies to conduct pediatric drug research. For example, regulations in some countries such as the United States or the European Union require pharmaceutical companies to submit Pediatric Investigation Plans (PIPs) when applying for new drug registration, which has significantly boosted pediatric medicine research in those countries. Studies show that the implementation of the EU Pediatric Regulation has led to significant progress in the development of pediatric formulations. For instance, in the case of cardiovascular drugs, there has been a clear trend of pediatric drugs shifting from liquid formulations to smaller solid (multiple granule) or orally disintegrating formulations since the regulation was enacted (Thabet et al., 2018). Similarly, these countries also offer incentives policies such as patent term extensions. Specifically, the US offers a pediatric exclusivity policy which grants drugmakers an additional six-month market exclusivity period for conducting successful pediatric research on drugs. However, studies found that this policy mainly promoted pediatric research for drugs that already had adult indications with limited impact on the development of new drugs specifically for children (Olson and Yin, 2018). This highlights that incentive policies alone cannot fully address the lack of R&D in pediatric drugs. Therefore, combining mandatory regulations with incentive policies may yield better outcomes for pediatric medicine R&D in China. While mandatory regulations directly encourage companies to conduct pediatric drug research, incentive policies can provide additional motivation. This dual approach could help address the current gaps in pediatric medicine development more effectively.

### 4.2 The synergy of policies for pediatric medicine needs to be strengthened

From the overall content of existing pediatric medicine policies, supply-oriented policies tools related to research and development, information support, and review and approval, etc., have developed rapidly and are quite comprehensive. However, environmental policy tools related to laws and regulations, medical insurance support and demand-oriented policy tools related to access and price are insufficient (Zhu et al., 2024). This imbalance in policy structure may compromise the comprehensiveness and coherence of pediatric medicine policies, thereby affecting their overall synergy.

This lack of policy synergy is evident in the following example: although the number of pediatric medicines in China has increased in recent years, there is a significant difference in the availability of pediatric medicines across different therapeutic areas (Wei et al., 2024; Wu et al., 2023). This indicates a need to further optimize the research and production of pediatric medicine, especially in therapeutic areas beyond traditional strengths. Additionally, regarding drug appropriateness, oral formulations in the “Encouraged Research and Submission List of Pediatric Medicine” often show poor suitability for dosage form in actual clinical practice. For example, in a certain tertiary children’s hospital, it is quite common for newborns and infants to be prescribed unsuitable dosage forms such as tablets or capsules (Xiao et al., 2024).

Furthermore, the original policy goal of national VBP is to provide patients with drugs of the same quality at lower prices. However, there is a discrepancy between the pediatric drugs included in VBP and the actual clinical needs in terms of variety, disease areas, suitable dosage forms and appropriate specifications. This leads to an imbalance in the supply of clinical pediatric medicines. Some suitable dosage forms and specifications for children are not included in VBP, further exacerbating the shortage of clinical pediatric medicines (Song et al., 2024; Liu et al., 2023).

### 4.3 The effectiveness of incentive policies still needs to be improved

Currently, the accessibility of pediatric medicine in China remains relatively low, especially for medicine intended for young children (Dai et al., 2020; Wei et al., 2019). Although policies such as trial data protection, market exclusivity and exclusive pricing rights are widely used internationally to incentivize the development of pediatric medicine, these policies have not been effectively implemented in China. This results in a lack of economic returns for companies to offset the enormous costs of research and development. Companies have low enthusiasm for pediatric medicine R&D. Studies have shown that financial support from public and charity is crucial for accelerating the development of pediatric drugs. The combination of financial and business models can overcome the lack of economic incentives and speed up the development of pediatric drugs (Das et al., 2018). Additionally, countries like the UK and Japan have implemented separate pricing incentive policies for pediatric drugs through drug value assessment to promote the development of pediatric medicine (Zhang et al., 2021; Ma et al., 2022). Moreover, pediatric extrapolation can reduce R&D costs, avoid duplicate trials, enhance efficiency and expedite drug approval to meet urgent clinical needs. In 2024, ICH supplemented E11(R1) with E11A, offering researchers a clear framework for disease and pharmacological evaluation, safety extrapolation and study design, ensuring the scientific rigor of extrapolation and supporting regulatory decisions. The development of virtual clinical trials provides new research avenues for pediatric medicine. The PBPK model helps researchers with drug dose selection, dosing regimen optimization and drug interaction assessment (Johnson et al., 2021), while virtual patient models and computer simulations enable early identification of promising treatment options, reducing reliance on animal and traditional clinical trials (Carlier et al., 2018). China still needs to continuously improve the policy framework of incentives to accelerate pediatric drug development and improve accessibility.

## 5 Conclusion

In this article, we review the progress and achievements of incentive policies related to pediatric medicine in China, highlighting the complexity and urgency of improving pediatric medicine accessibility. While significant progress has been made, policy synergy and effectiveness in R&D, approval, production, access, payment and use still need enhancement. We suggest policymakers adopt more effective international mandatory regulations or incentive policies and strengthen policy coordination. Integrating fiscal and medical insurance tools can further boost accessibility. Additionally, increasing economic incentives and value assessments for pediatric medicine can motivate pharmaceutical companies to invest in R&D, ensuring the safety, efficacy and affordability of pediatric drugs.

## Data Availability

The datasets presented in this study can be found in online repositories. The names of the repository/repositories and accession number(s) can be found in the article/Supplementary Material.
