# Supplementary material for: Reform progress and achievements of China’s incentive policies for pediatric medicine over the last decade
Source: Front Pharmacol. 2025 Mar 26;16:1561095. doi: 10.3389/fphar.2025.1561095 (PMC11980170; doi:10.3389/fphar.2025.1561095)
Supplement: Supplementary file 1 [file Table1.docx]

| Supplementary Material  Supplementary document table 1 Some significant policies on pediatric medication in China in recent years | | | | | |
| --- | --- | --- | --- | --- | --- |
| **Drug Development Stage** | **Key information related to**  **pediatric medication** | **Issuing Institution** | **Release Date** | **Document Name** | **Document Content** |
| Research and Development phase | Increase financial support for pediatric drug research and development | Ministry of Science and Technology | 2017.01 | Notice on Organizing the Application for the Major Project for New Drug Innovation in 2017 | Includes pediatric drug varieties and key technology research and development in the special project support, and prioritizes the varieties in the First Batch of Encouraged Research and Submission List of Pediatric Medicine |
|  |  | Ministry of Science and Technology, China Food and Drug Administration (CFDA) | 2018.01 | Guidelines on Strengthening and Promoting Food and Drug Science and Technology Innovation | Focuses on supporting pediatric-specific drug research and development through relevant national science and technology plans (special projects, funds, etc.) |
|  | Improve the protection of intellectual property rights for children's medicines | State Council | 2017.10 | Opinions on Deepening the Review and Approval System Reform to Encourage Drug and Medical Device Innovation | Provide a certain period of data protection for pediatric-specific drug applicants who submit their own undisclosed test data and other data |
|  |  | National Medical Products Administration (NMPA) | 2018.04 | Implementation Measures for Drug Trial Data Protection (Interim) (Draft for Comments) | Provides a 6-year data protection period for pediatric drugs |
|  |  | National Medical Products Administration (NMPA) | 2022.05 | Regulations on the Implementation of the People's Republic of China Drug Administration Law (Revised Draft for Comments) | For the first approved pediatric-specific new varieties, dosage forms, and specifications, as well as those that add pediatric indications or dosage and administration instructions, a market exclusivity period of up to 12 months is granted. During this period, no identical varieties are approved for market. |
|  | Technical guidelines for pediatric medicines development | Center for Drug Evaluation (CDE)，National Medical Products Administration | 2014.07 | Technical Guidelines for Pharmacokinetic Studies in Pediatric Populations | Standardizes and guides pharmacokinetic studies in pediatric populations |
|  |  |  | 2016.03 | Technical Guidelines for Clinical Trials in Pediatric Populations | Standardizes clinical trials in pediatric populations in China to ensure the effectiveness and safety of pediatric drug |
|  |  |  | 2017.03 | Technical Guidelines for Non-clinical Safety Studies of Pediatric Drugs (Draft for Comments) | Clarify and unify the technical requirements for non-clinical safety evaluation of pediatric drugs to better guide research and evaluation work |
|  |  |  | 2017.05 | Technical Guidelines for Extrapolation of Adult Drug Data to Pediatric Populations | Make the most of existing data, reduce unnecessary pediatric studies, and use data extrapolation to improve and enrich drug information for pediatric populations in instructions, guiding clinical drug use |
|  |  |  | 2020.08 | Technical Guidelines for Real-World Studies Supporting Pediatric Drug Research and Review (Trial) | Guide and standardize the use of real-world evidence to support pediatric drug development and review |
|  |  |  | 2020.12 | Technical Guidelines for Clinical Pharmacology Research of Pediatric Drugs | Clarifies the technical requirements for clinical pharmacology of pediatric drugs, promoting pediatric drug research and development |
|  |  |  | 2020.12 | Technical Guidelines for the Development of Pediatric Drugs (Chemical Drugs) (Trial) | Discuss the characteristics of pediatric drug pharmaceutical development from aspects such as the selection of administration routes and dosage forms, active ingredients, excipients, packaging systems, and drug delivery devices, patient acceptability, etc., providing research ideas and technical guidance for pediatric drug pharmaceutical development |
|  |  |  | 2021.09 | Technical Guidelines for Clinical Trials of Attention Deficit Hyperactivity Disorder (ADHD) Drugs (Trial) | Encourage and promote ADHD drug research and development, standardize clinical study design, and provide technical specifications for reference |
|  |  |  | 2021.09 | Technical Guidelines for Clinical Trial of Pediatric Chemical Modified New Drugs (Trial) | Elaborate on the clinical study design of common modified new drugs and point out issues that need attention |
|  |  |  | 2022.01 | Technical Guidelines for Clinical Trial of Drugs for Pediatric Pulmonary Arterial Hypertension | Guide clinical trials for pediatric pulmonary arterial hypertension and provide technical specifications for reference |
|  |  |  | 2022.10 | Technical Guidelines for Design and Evaluation of Taste of Pediatric Drugs (Trial) | Further clarifies the relevant research requirements for the design and evaluation of the taste of pediatric drugs |
|  |  |  | 2023.03 | Technical Guidelines for Clinical Research and Development of Pediatric Anticancer Drugs | Provides ideas and technical suggestions for the clinical research and development of new pediatric anticancer drugs |
|  |  |  | 2023.03 | Technical Guidelines for the Application of Physiologically Based Pharmacokinetic (PBPK) Models in Pediatric Drug Development | Provide general suggestions on the construction, evaluation, and application of PBPK models in the drug development process for pediatric populations |
|  |  |  | 2023.04 | Technical Guidelines for Quantitative Extrapolation Methods of Adult Drug Data to Pediatric Populations (Trial) | Provides guidance on extrapolation strategies, main quantitative methods, and application scenarios based on existing adult and other data extrapolated to pediatric populations, thereby providing guidance for pediatric drug research and development based on adult drug data extrapolation for registration purposes |
|  |  |  | 2024.02 | Technical Guidelines for Clinical Research and Development of Traditional Chinese Medicine New Drugs for Pediatric Constipation (Trial) | Encourages the research and development of pediatric traditional Chinese medicine, guides applicants to develop pediatric constipation treatment-related traditional Chinese medicine new drug that meet the advantages and characteristics of traditional Chinese medicine according to the “three combinations” evidence system for registration review |
|  |  |  | 2024.06 | Technical Guidelines for Pediatric Clinical Trial Pharmacovigilance (Draft for Comments) | Guides a scientific, standardized, and replicable model of pharmacovigilance in pediatric clinical trials |
|  | Improve service and support capabilities | Center for Drug Evaluation (CDE)，NMPA | 2022.06 | Administrative Procedures for Pediatric Drug Communication Applications and Management (Draft for Comments) | Improves the quality and efficiency of pediatric drug communication |
|  |  |  | 2023.04 | Administrative Rules for Application and Management of Class I Meetings in Pediatric Drug Communication (Trial) | Refines the application and management process of pediatric drug communication, strengthens the management of pediatric drug communication, improves communication efficiency, and effectively promotes the innovation and research and development of pediatric drugs in China |
|  | Encouraged Research and Submission List of Pediatric Medicine | National Health and Family Planning Commission, Ministry of Industry and Information Technology, China Food and Drug Administration | 2016.06 | Notice on Issuing the First Batch of Encouraged Research and Submission List of Pediatric Medicine | Promote the research and development, review and approval of pediatric appropriate varieties, dosage forms, and specifications in the form of an encouragement list to meet the clinical drug needs of pediatrics |
|  |  | National Health and Family Planning Commission, Ministry of Industry and Information Technology, China Food and Drug Administration | 2017.06 | Notice on Issuing the Second Batch of Encouraged Research and Submission List of Pediatric Medicine |  |
|  |  | National Health Commission, Ministry of Industry and Information Technology, National Medical Products Administration | 2019.07 | Notice on Issuing the Third Batch of Encouraged Research and Submission List of Pediatric Medicine |  |
|  |  | National Health Commission, Ministry of Science and Technology, Ministry of Industry and Information Technology, National Medical Products Administration | 2023.08 | Notice on Issuing the Fourth Batch of Encouraged Research and Submission List of Pediatric Medicine |  |
|  |  | National Health Commission, Ministry of Science and Technology, Ministry of Industry and Information Technology, National Medical Products Administration | 2024.08 | Notice on Issuing the Fifth Batch of Encouraged Research and Submission List of Pediatric Medicine |  |
| Review and Approval phase | Accelerate the review and approval of pediatric drugs | State Council | 2015.08 | Opinions on Reforming the Drug and Medical Device Review and Approval System | Accelerate the review and approval of pediatric drugs |
|  |  | China Food and Drug Administration | 2015.11 | Announcement on Several Policies of Drug Registration, Review and Approval by the China Food and Drug Administration (No. 230 of 2015) | Implement separate queuing for pediatric drug registration applications to accelerate review and approval |
|  |  | Center for Drug Evaluation (CDE)，NMPA | 2016.01 | Announcement on the Basic Principles for the Evaluation of Priority Review and Approval of Urgently Needed Pediatric Drugs and the First Batch of Priority Review Drugs | Release the basic principles for the priority review and approval of clinically urgent pediatric-specific drugs |
|  |  | National People's Congress (NPC) | 2019.8 | Drug Administration Law of the People's Republic of China (2019 Revision) | Encourage the research and innovation of pediatric drugs, support the development of new varieties, dosage forms, and specifications of pediatric drugs that meet the physiological characteristics of children, and give priority to the review and approval of pediatric drugs |
|  |  | National People's Congress (NPC) | 2019.12 | Basic Medical and Health Promotion Law of the People's Republic of China | Establishes and improves a drug review and approval system oriented by clinical needs, supports the research, production, and supply of pediatric drugs, and meets the needs of disease prevention and treatment. |
|  |  | National Medical Products Administration | 2020.08 | Administrative Procedures for Priority Review and Approval of Drug Marketing Authorization (Trial) | It stipulates that priority review and approval shall be given to children's drugs that are clearly in short supply in the market and that are encouraged to be submitted for research and development |
|  |  | National Medical Products Administration | 2022.05 | Regulations on the Implementation of the Drug Administration Law of the People's Republic of China (Revised Draft for Comments) | Encourage the research and innovation of pediatric drugs, support drug holders to develop new varieties, new dosage forms, and new specifications of pediatric drugs, and give priority to the review and approval of pediatric drugs. Strengthen communication with applicants during drug research and development and registration to promote the accelerated launch of pediatric drugs to meet the clinical needs |
| Production and Supply phase | Ensures the production and supply of pediatric drugs. | National Health and Family Planning Commission , National Development and Reform Commission (NDRC) , Ministry of Education , Ministry of Finance , Ministry of Human Resources and Social Security , National Administration of Traditional Chinese Medicine | 2016.05 | Opinions on Strengthening the Reform and Development of Pediatric Medical and Health Services | Ensures the supply and security of pediatric drugs. Establishes and improves an early warning mechanism, timely grasps the production dynamics of shortage pediatric drugs, and resolves prominent problems and difficulties of manufacturers, and improves production and supply capabilities. |
|  |  | Ministry of Industry and Information Technology , National Development and Reform Commission, Ministry of Science and Technology, Ministry of Commerce, National Health and Family Planning Commission, China Food and Drug Administration | 2016.11 | Guiding Opinions on the Development Plan of the Pharmaceutical Industry | Comprehensively use policies such as supervision, medical insurance, pricing, procurement, and use to guide enterprises to develop and produce short drugs, focusing on solving the lack of pediatric drugs and the weak supply of low-priced pediatric drugs |
|  |  | State Council | 2021.5 | Key Tasks for Deepening the Reform of the Medical and Health System in 2021 | Strengthen the supply and security of pediatric drugs |
|  |  | State Council | 2021.09 | China's Children's Development Outline (2021-2030) | Improve the diagnosis and treatment system, drug supply system, and comprehensive security system for children's hematological diseases, malignant tumors and other serious diseases |
| Access phase | Improve the access to procurement and admission in medical institutions | General Office of the State Council | 2015.02 | Guiding Opinions on Improving the Volume-Based Procurement of Drugs Use in Public Hospitals | Non-patent drugs for women and children are not included in the volume-based procurement and are purchased directly through online. |
|  |  | National Health and Family Planning Commission | 2015.09 | Notice on Further Strengthening the Provision and Use of Pediatric Drugs in Medical Institutions | Non-patent drugs for women and children are not included in the volume-based procurement and are purchased directly to meet clinical needs through online. |
|  |  | National Health Commission , National Administration of Traditional Chinese Medicine | 2022.07 | Notice on Further Strengthening Drug Use Safety Management and Improving the Level of Rational Drug Use | For pediatric drugs with clear pediatric indications and dosage and administration instructions, hospitals can increase the scope of medicine without being limited by the "one drug, two specifications" and the total number of drug varieties |
| Payment phase | Expand the scope of medical insurance payment | National Health and Family Planning Commission | 2014.05 | Several Opinions on Ensuring the Use of Children's Medicine | The role of medical insurance in ensuring pediatric medicine should be leveraged by timely include pediatric-appropriate dosage forms and specifications in the basic medical insurance payment scope |
|  |  | National Health Commission | 2019.07 | Notice on Carrying Out Medical Treatment and Security Management for Pediatric Blood Diseases and Malignant Tumors | Improve the dynamic adjustment mechanism of the medical insurance drug directory and gradually include more eligible drugs for the treatment of severe diseases such as pediatric blood diseases and malignant tumors into the medical insurance payment scope |
|  |  | National Healthcare Security Administration | 2020.08 | National Medical Insurance Drug Directory Adjustment Work Plan for 2020 | Drugs on the Encouraged Research and Submission List of Pediatric Medicine can be included in the scope of the medical insurance drug directory |
| Usage phase | Strengthen the management of children's drug instruction | CDE, NMPA | 2021.09 | Technical Guidelines for Writing Pediatric Drug Instructions of Chemical Drugs and Therapeutic Biological Products | Promote enterprises to orderly draft and improve pediatric drug instructions, better guiding the rational use of drugs in clinical practice |
|  |  | CDE, NMPA | 2023.05 | Work Procedures for Adding Pediatric Drug Information in Instructions of Marketed Drugs (Trial) | Promote the resolution of the widespread issue in China's pediatric clinical practice of lagging supplementation or revision of pediatric drug information in instructions |
|  | Regulate the prescription of children's medicines | National Health and Family Planning Commission | 2015.08 | Notice on Strengthening the Equipping and Use of Pediatric Drugs in Medical Institutions | All medical institutions should refer to the national prescription collection, basic drug clinical application guidelines, and prescription collection to standardize prescription behavior, promote the informatization of drug management, and improve the level of rational drug use |
|  |  | State Council | 2016.04 | Key Tasks for Deepening the Reform of the Medical and Health System in 2016 | Study the suitable dosage forms and specifications of basic pediatric drugs, strengthen the clinical application and prescription training of basic pediatric drugs, and increase pharmaceutical services in impoverished areas in China |
|  | Improve the comprehensive evaluation capacity of pediatric drug clinical use | National Health Commission, National Administration of Traditional Chinese Medicine | 2019.01 | Notice on Strengthening the Management of Basic Drug Equipping and Use in Public Medical Institutions | Prioritize the compilation of work plans for pediatric drugs, establish evaluation bases, carry out comprehensive clinical evaluations, and promote comprehensive application of evaluation results |
|  |  | National Health Commission Development Research Center | 2021.12 | Technical Guidelines for Comprehensive Clinical Evaluation of Pediatric Drugs (2021 Edition) | Focus on the actual problems in the clinical use and evaluation of pediatric drugs in China, refer to the beneficial practices and experiences of international pediatric drug evaluation, and gradually achieve the scientification, homogenization, and standardization of the national comprehensive evaluation of pediatric drugs |
